# Atrial fibrillation in breast cancer therapy: does tamoxifen confer a lower risk than aromatase inhibitors?

**DOI:** 10.1186/s40959-025-00352-3

**Published:** 2025-05-31

**Authors:** Patrick A. Kwaah, Samuel A. Mensah, Emmanuel A. Agyemang, Joseph S. Kekrebesi, Daniil Katkov, Abraham Carboo, Grace Appah, Hamza A. Rashid, Jennifer M. Kwan

**Affiliations:** 1 Department of Internal Medicine, Yale School of Medicine, 64 Robbins St, Waterbury, CT 06708 USA; 2 Department of Internal Medicine, West Virginia University, Morgantown, WV USA; 3 Department of Internal Medicine, Newark Beth Israel Medical Center, Newark, USA; 4 Division of Cardiology, Emory University School of Medicine, Atlanta, Georgia; 5 Section of Cardiovascular Medicine, Yale School of Medicine, New Haven, CT USA

## Abstract

**Introduction:**

Aromatase inhibitors (AIs) have been linked to increased atrial fibrillation(AF) risk due to estrogen depletion however tamoxifen's effect on AF remains conflicting. This study investigates the risk of AF associated with AI use compared to tamoxifen in breast cancer patients.

**Methods:**

A retrospective cohort analysis was conducted using the TriNetX database from 2015 to 2024. Breast cancer patients were categorized into two groups: AI users (anastrozole, exemestane, or letrozole) and tamoxifen users. A propensity score matching (1:1) adjusted for demographics, comorbidities, concurrent therapies, and lab values. The incidence of AF was assessed at 1, 5, and 10-years post-treatment initiation.

**Results:**

The study included 220,552 AI users and 73,388 tamoxifen users before matching, with 54,175 patients in each group after matching. At 1 year, AI users had a higher risk of AF (0.5% vs. 0.4%, RR: 1.36, *p* = 0.001). At 5 years, AF incidence remained higher in the AI group (1.2% vs. 1.1%, RR: 1.13, *p* = 0.035).However at 10 years, the difference in AF risk between the two groups was no longer significant (1.6% vs. 1.5%, RR: 1.05, *p* = 0.295).

**Conclusion:**

AI use is associated with a higher risk of AF than tamoxifen in the first 5 years of treatment, but the risk equalizes at 10 years. Long-term hormonal therapy has an increased risk of AF, highlighting the need for ongoing monitoring and management of risk factors.

## Introduction

Atrial fibrillation (AF) is the most prevalent cardiac arrhythmia, affecting an estimated 59 million people worldwide in 2019 and contributing to over 300,000 deaths [1]. Aromatase inhibitors (AIs) and tamoxifen, a selective estrogen receptor modulator, are two effective hormonal therapies commonly used in the treatment of breast cancer, which have been associated with increased risk of AF [2, 3]. Anti-estrogens inhibit estrogen's regulation of cardiac electrical conduction and myocardial contractile function, potentially increasing this risk [4].

AIs, preferred in postmenopausal women, inhibit the conversion of androgen to estrogen reducing estrogen levels and slows breast cancer growth [5]. Tamoxifen on the other hand is favored in premenopausal women and blocks estrogen binding in breast tissue, leading to cancer cell death [6]. Previous studies have shown an increased risk of cardiac arrhythmias, particularly AF, with the use of AIs and have recommended increased surveillance to guide timely anticoagulation therapy. The reduced estrogen level with AI use has been proposed as the mechanism for this increased risk [3, 7]. However, data on tamoxifen’s effects on the risk of AF are conflicting. While some studies have suggested an increased risk of AF, others have demonstrated a decreased risk [7–9]. Therefore, we sought to explore the risk of AF associated with AI use compared to tamoxifen use in breast cancer patients.

## Methods

We conducted a retrospective observational 1:1 propensity-matched cohort analysis of individuals 18 years and above using the TriNetX Analytics Research Network between 01/01/2015 and 12/31/2024. Data curation was done on 02/09/2025. The study adhered to the STROBE guidelines for observational studies. The TriNetX database contains aggregated statistics and de-identified data without protected health information; therefore, it was exempt from Institutional Review Board (IRB) review. Breast cancer patients were identified using International Classification of Diseases, 10 th Revision (ICD-10) diagnostic code C50 and the population was stratified into two cohorts: 1) patients with breast cancer on AIs (anastrozole or exemestane or letrozole) but not on tamoxifen and 2) Patients with breast cancer on tamoxifen but not on AIs. Patients with a history of ventricular fibrillation or other arrythmias (ICD-10 I49, I48.3 and I74) were excluded at baseline. Cohorts were matched using propensity score matching (PSM) with a nearest neighbor algorithm and a 0.2 standard deviation caliper width to adjust for confounders. Matching was based on demographics (age, sex, menopausal status), comorbidities (hypertension, diabetes, dyslipidemia, ischemic heart disease, heart failure, other malignancies), concurrent therapy (anthracycline use, steroids, radiation exposure, concurrent medications), and laboratory values (Table 1). We compared Cohorts 1 and 2, exploring the primary outcome of AF at 1 year, 5 years and 10 years after the initiation of AIs and tamoxifen in the respective cohorts. Risk ratios were calculated with statistical significance set at a two-sided *p*-value of < 0.05. All analyses were performed on the TriNetX online platform. Sex data was missing for 2.9% and 1.6% in the overall cohort before PSM for the AI group and tamoxifen group respectively. This is likely due to incomplete documentation or de-identification protocols. IRB approval was not required due to the use of de-identified data.

**Table 1:** Comparative Analysis for Aromatase Inhibitors vs Tamoxifen

	**Before Propensity Score Matching**			**After Propensity Score Matching**		
**Characteristic**	**Aromatase Inhibitors group *n* = 220,552**	**Tamoxifen group *n* = 73,388**	**Standard Difference**	**Aromatase Inhibitors group *n* = 54,175**	**Tamoxifen group *n* = 54,175**	**Standard Difference**
**Demographics**						
Age, mean ± SD (years)	65.5 ± 11.1	54.4 ± 12.9	0.919	57.7 ± 12.4	57.7 ± 12.7	0.004
Female	96.9%	95.9%	0.053	97.4%	96.7%	0.041
Male	0.2%	2.5%	0.198	0.8%	1.4%	0.056
**Race**						
White race	67.2%	59.7%	0.156	62.3%	63.3%	0.021
Black or African American	10.8%	9.1%	0.059	8.9%	9.6%	0.023
Asian	4.8%	9.3%	0.176	8.0%	7.2%	0.029
Other race	4.6%	7.6%	0.128	7.3%	6.3%	0.038
**Comorbidities**						
Hypertensive diseases	39.0%	21.6%	0.384	24.8%	25.9%	0.024
Ischemic heart diseases	7.6%	3.6%	0.172	4.4%	4.4%	0.004
Heart failure	3.7%	1.6%	0.133	2.2%	1.9%	0.022
Other Peripheral vascular diseases	2.3%	1.4%	0.068	1.6%	1.6%	0.001
Other Venous embolism and thrombosis	2.9%	1.2%	0.116	1.8%	1.5%	0.022
Diabetes mellitus	15.0%	7.6%	0.235	9.0%	9.1%	0.006
Thyrotoxicosis	1.8%	1.6%	0.013	1.7%	1.7%	0.005
Overweight and obesity	16.3%	10.8%	0.164	11.7%	12.5%	0.024
Disorders of lipoprotein metabolism and other lipidemia	32.4%	17.8%	0.340	20.6%	21.4%	0.020
Sleep disorders	12.7%	9.2%	0.110	9.9%	10.3%	0.014
Other chronic obstructive pulmonary disease	4.5%	2.1%	0.136	2.5%	2.6%	0.005
Malignant neoplasm of corpus uteri	1.0%	0.5%	0.056	0.6%	0.6%	0.007
Malignant neoplasm of ovary	0.6%	0.4%	0.028	0.5%	0.5%	0.001
Neoplasm of uncertain behavior of other and unspecified sites	4.3%	3.9%	0.022	3.9%	3.9%	0.002
Chronic kidney disease	5.3%	2.5%	0.145	3.2%	2..9%	0.018
Fibrosis and cirrhosis of liver	0.8%	0.5%	0.041	0.6%	0.6%	0.006
Nicotine dependence	6.1%	4.5%	0.074	4.5%	5.2%	0.029
Alcohol-related disorders	1.3%	1.2%	0.016	1.1%	1.3%	0.014
Menopausal and female climacteric states	5.7%	5.2%	0.024	5.7%	6.0%	0.011
Primary ovarian failure	0.9%	0.6%	0.039	0.8%	0.7%	0.009
Asymptomatic menopausal state	12.3%	5.4%	0.246	7.0%	7.2%	0.007
**Procedure**						
Radiation Oncology Treatment	16.2%	14.7%	0.040	14.3%	15.2%	0.025
**Laboratory Test**						
Potassium mmol/L	4.1 ± 0.4	4.1 ± 0.4	0.062	4.1 ± 0.4	4.1 ± 0.4	0.012
Creatinine (mg/dL)	0.9 ± 1.0	0.8 ± 1.7	0.028	0.8 ± 0.5	0.8 ± 1.2	0.016
Cholesterol in LDL (mg/dL)	107.1 ± 37.1	104.8 ± 34.1	0.064	110.6 ± 36.3	105.0 ± 35.1	0.175
C-reactive protein (mg/L)	22.0 ± 46.6	16.4 ± 37.0	0.130	21.1 ± 46.2	17.1 ± 38.3	0.065
Thyroxine (T4, ug/dL)	8.0 ± 2.1	8.1 ± 2.2	0.074	8.0 ± 2.2	8.1 ± 2.2	0.076
Hemoglobin A1c (%)	6.2 ± 1.5	5.9 ± 1.3	0.227	6.1 ± 1.4	6.0 ± 1.4	0.040
Corrected QT Interval (QTc)	433.4 ± 28.1	432.1 ± 26.6	0.046	431.6 ± 27.7	431.8 ± 27.3	0.004
BMI	29.4 ± 6.9	27.7 ± 6.7	0.255	28.9 ± 6.8	28.1 ± 6.8	0.118
**Medications**						
Beta Blockers	28.8%	18.5%	0.225	20.6%	20.5%	0.003
ACE Inhibitors	17.0%	10.9%	0.179	11.9%	12.3%	0.014
Diuretics	25.3%	15.1%	0.256	17.3%	17.6%	0.008
Antilipemic Agents	31.1%	15.1%	0.386	18.3%	18.7%	0.010
Antiarrhythmics	47.7%	43.1%	0.094	42.8%	43.6%	0.016
Calcium Channel Blockers	16.8%	8.7%	0.245	10.4%	10.3%	0.004
Antineoplastics, Alkylating Agents	9.1%	12.3%	0.106	10.7%	10.9%	0.005
Leuprolide	1.2%	0.9%	0.032	1.0%	1.1%	0.008
Goserelin	0.9%	0.8%	0.012	0.9%	1.0%	0.013
Fulvestrant	0.8%	0.2%	0.083	0.5%	0.3%	0.023
Immunological Agents, Other	11.0%	9.4%	0.053	9.7%	9.8%	0.004
Platelet Aggregation Inhibitors	20.7%	12.7%	0.216	14.4%	14.5%	0.004
Anticoagulants	31.2%	24.5%	0.149	25.9%	25.7%	0.003
Glucocorticoids	53.9%	52.4%	0.030	51.1%	52.1%	0.020
Alendronate	4.3%	2.5%	0.096	3.2%	3.3%	0.004
Zoledronic Acid	2.3%	1.2%	0.080	1.6%	1.5%	0.007
Levothyroxine	13.9%	9.4%	0.139	10.7%	10.7%	0.001
Non-Steroidal Anti-Inflammatory Analgesics	18.8%	20.6%	0.045	19.8%	20.2%	0.008

*N* number, *SD* Standard deviation, *ACE* Angiotensin converting enzyme, *LDL* Low density lipoprotein, *BMI* Body mass index

Significance is when *p*-value < 0.05

## Results

The initial cohort included 220,552 AI users and 73,388 tamoxifen users. After PSM, 54,175 patients remained in each group. The mean age was 57.7 ± 12.4 years in the AI group and 57.7 ± 12.7 years in the tamoxifen group. Demographics, comorbidities, and laboratory values were generally well-balanced after PSM, with nearly all standardized differences below 0.1. Notable exceptions included BMI and LDL cholesterol, which had standardized differences of 0.118 and 0.175, respectively (Table 1) [10]. Comorbidities were comparable between groups, including hypertension (24.8% vs. 25.9%), diabetes mellitus (9.0% vs. 9.1%), chronic kidney disease (3.2% vs. 2.9%), and malignant uterine and ovarian neoplasm. Menopausal status was also well-balanced, with a standardized difference of 0.011. Radiation therapy and concurrent hormonal and antineoplastic medications were similar between the groups (Table 1). A total of 1,167 patients in the AI group and 1,048 in the tamoxifen group were excluded because they had AF before initiation of the respective medications. At 1 year (mean follow-up: AI, 314.5 days; tamoxifen, 320.0 days), 0.5% of AI users developed AF compared to 0.4% of tamoxifen users (RR: 1.36, 95% CI: 1.12–1.64, *p* = 0.001). At 5 years (mean follow-up: AI, 1044.6 days; tamoxifen, 1138.8 days), AF incidence was 1.2% in the AI group and 1.1% in the tamoxifen group (RR: 1.13, 95% CI: 1.01–1.27, *p* = 0.035). At 10 years (mean follow-up: AI, 1317.9 days; tamoxifen, 1466.1 days), AF incidence was 1.6% in the AI group and 1.5% in the tamoxifen group (RR: 1.05, 95% CI: 0.96–1.16, *p* = 0.295), indicating no significant difference. The rate and relative risk of AF in both the AI and tamoxifen groups before PSM are presented in Table 2.

**Table 2:** Atrial fibrillation outcome after follow-up before and after propensity score matching

	**Before propensity score matching**				**After propensity score matching**			
	Rate of AF in AI group *N* = 220,552	Rate of AF in Tamoxifen group *N* = 73,388	Relative risk	*P* value	Rate of AF in AI Group *N* = 54,175	Rate of AF in Tamoxifen group *N* = 54,175	Relative risk	*P* value
1 year	0.008	0.003	2.87 (2.46, 3.35)	< 0.01	0.005	0.004	1.36 (1.12, 1.64)	0.001
5 years	0.020	0.008	2.57 (2.35, 2.82)	< 0.01	0.012	0.011	1.130 (1.01, 1.27)	0.035
10 years	0.026	0.011	2.31 (2.14, 2.49)	< 0.01	0.016	0.015	1.05 (0.96, 1.16)	0.295

*AF* Atrial fibrillation, *AI* Aromatase inhibitor, *N* Number

Before propensity score matching: 7,871 patients in Cohort 1 and 1,073 patients in Cohort 2 were excluded from results because they had the outcome prior to the time window

After propensity score matching: 1,167 patients in Cohort 1 and 1,048 patients in Cohort 2 were excluded from results because they had the outcome prior to the time window

## Discussion

Our study found an increased risk of new onset AF with AI use compared to tamoxifen use in breast cancer patients on receiving hormonal therapy. However, the relative risk gradually decreased over time, with no observed difference at 10 years. Our findings align with previous studies that reported a higher risk of new-onset AF in patients on AI compared to patients on tamoxifen [7]. Furthermore, our results are consistent with Veronesi et al., who reported an increased risk of AF after 11 years of tamoxifen use [9].

Aromatase inhibitors have been associated with an increased risk of AF due to the depletion of estrogen levels [11]. The loss of estrogen’s cardioprotective effects, metabolic alterations, and direct impact on cardiac electrophysiology through cardiac ion channels and autonomic regulation contribute to the heightened risk of AF [11–13]. On the other hand, the underlying mechanism of tamoxifen’s effect on AF remains unclear. Animal studies have demonstrated that tamoxifen can block the delayed rectifier potassium current in cardiac myocytes, potentially leading to QT interval prolongation and an increased risk of arrhythmia [14]. However, other studies have reported that chronic tamoxifen use upregulates various potassium channels, including the Ca^2^⁺-independent transient outward, ultrarapid delayed rectifier, steady-state, and inward rectifier K⁺ currents, which may stabilize the cardiac action potential and reduce the likelihood of arrhythmias [15]. Additionally, some researchers postulate that the anti-inflammatory properties of tamoxifen may contribute to cardiac environment stabilization, thereby reducing the propensity for arrhythmias [16].

Extended tamoxifen treatment for 10 years has been demonstrated to significantly reduce breast cancer recurrence rates, a recommendation endorsed by the American Society of Clinical Oncology (ASCO) in its 2019 Clinical Practice Guideline Focused Update [17, 18]. Our findings are therefore crucial, as an increasing number of hormone-responsive breast cancer patients are being treated with prolonged tamoxifen therapy. Randomized controlled trials are needed to compare these risks and validate our observations.

The integration of mobile and wearable health technologies into oncologic care—particularly for breast cancer patients receiving endocrine therapy—represents a promising advancement in longitudinal monitoring [19]. Devices such as smartwatches and biosensor-enabled monitors are increasingly used for real-time, non-invasive tracking of cardiovascular parameters, including heart rate variability and arrhythmia detection [19, 20]. This is especially relevant for patients treated with aromatase inhibitors or long-term tamoxifen, which are associated with an elevated risk of arrhythmia, including AF [7, 9]. Wearable technologies allow for early detection of subclinical cardiotoxicity, enabling timely clinical intervention and potentially reducing long-term morbidity. Their continuous monitoring capabilities also support remote patient engagement, adherence to therapy, and data-driven clinical decision-making which are key components of effective survivorship care [21, 22].

### Limitations

The TriNetX database aggregates data from multiple healthcare organizations but may miss outcomes outside these systems. Despite propensity score matching, residual confounding persists due to the retrospective design. While menopausal status was adjusted, we were unable to account for differences in estrogen levels between cohorts. Additionally, medication dosage and adherence were not accounted for in the analysis.

## Conclusion

AI use was associated with a higher risk of new onset AF compared to tamoxifen up to 5 years, but no significant difference is observed at 10 years. There is therefore a risk of AF in breast cancer patients with long-term exposure to hormonal therapy that warrants vigilant long-term surveillance and risk factor optimization.

## Data Availability

No datasets were generated or analysed during the current study.
